# Supplementary material for: Functional Characterization of Splice Variants in the Diagnosis of Albinism
Source: Int J Mol Sci. 2024 Aug 8;25(16):8657. doi: 10.3390/ijms25168657 (PMC11355033; doi:10.3390/ijms25168657)
Supplement: Supplementary file 1 [file ijms-25-08657-s001.zip › Legends to Supplementary Figures.pdf]

Legends to Supplementary Figures

SUPPLEMENTARY FIGURE S1: Prediction of the effect on protein sequence of variants causing in frame or out of frame insertion of amino acid stretches.

The Expasy-translate tool (https://web.expasy.org/translate/) was used. A) *OCA2* c.2433-22889T>A; p.(Leu812Trpfs17*); B) *OCA2* c.2080-158A>G p.(Glu693_Ala694ins41); C) TYRP1 c.415G>A; p.(Val129Glyfs23*); D) *TYRP1* c.913+2T>G; p.(Glu237Alafs80*); E) *HPS1* c.1599-16T>G; p.Ala170Profs75* "- " indicates the next termination of translation.

SUPPLEMENTARY FIGURE S2: Functional analysis of Patients 9, 10 and 11 variants.

Patients 9 and 10 have respectively *OCA2* variant NM_000275.3:c.2080-20342A>G (intron 19) and *TYR* variant NM_000372.5:c.1185-27463T>G (intron 3). Among the algorithms used to assess splicing, only RNA-Splicer predicted an effect for these variants. Predictions were low for the *OCA2* variant c.2080-20342A>G (0.9897) and moderate for the *TYR* variant c.1185-27463T>G (0.9648). As these variants were very rare in GnomAD and found *in trans* to a pathogenic variant, we tested them using the minigene assay. RT-PCR products of the variant vectors were the same as those generated by the wild type vectors (band at 263 bp) (agarose gels not shown). Thus, these variants did not alter splicing of pre-mRNA of *OCA2* and *TYR*.

Patient 11 has *OCA2* variants NM_000275.3:c.2433-22889T>A (same as Patient 2) and NM_000275.3:c.1951+605G>T (intron 18) (absent from gnomAD3.2) predicted by RNA Splicer to include a 149 bp long pseudoexon. The minigene assay showed identical patterns with the variant and wild type vectors in HeLa cells. The 149 bp predicted pseudoexon was not observed. Instead, a 97 bp band was observed in both the variant and the wild type constructs, that upon sequencing corresponded to part of the intronic segment inserted in the construct (agarose gels and Sanger sequencing not shown). The same result was observed after transfection in MNT1 cells that were tested as a more "physiological" cell line than HeLa cells. This 97 bp band is therefore considered non-specific. In total, we do not have evidence that the NM_000275.3:c.1951+605G>T variant is pathogenic, and it remains a VUS at this point.

Presentation of the data is the same for the three variants. Top: a schematic representation of the minigene constructs in vector pSPL3B is shown, with the predicted pseudoexon (PE) as a red box. Bottom: schematic representation of the two RT-PCR products, with or without the pseudoexon. Blue arrows or arrowheads represent the primers used.

SUPPLEMENTARY FIGURE S3: Functional analysis of *AP3D1* variants in Patient 12.

Presentation of the data is the same for the two variants, NM_001261826.3:c.867G>T in exon 10 and NM_001261826.3:c.3486G>A in exon 31. A) Schematic representation of the exon in which the variant is located, with flanking exons. The variant is indicated in red. B) Schematic representation of the mRNA segment amplified by RT-PCR. Black arrows represent the primers. The size of the wild type amplified fragment is indicated in bp. (agarose gels not shown). C) Sanger sequencing of the RT-PCR product from the patient and one of the controls. Location of the variant is indicated. Heterozygosity for the variant indicates that both alleles of the patient are amplified, thus demonstrating normal splicing of the variant allele.
