# Supplementary material for: Functional Characterization of Splice Variants in the Diagnosis of Albinism
Source: Int J Mol Sci. 2024 Aug 8;25(16):8657. doi: 10.3390/ijms25168657 (PMC11355033; doi:10.3390/ijms25168657)
Supplement: Supplementary file 1 [file ijms-25-08657-s001.zip › Supplementary Figure S1.pptx]

## Slide 1
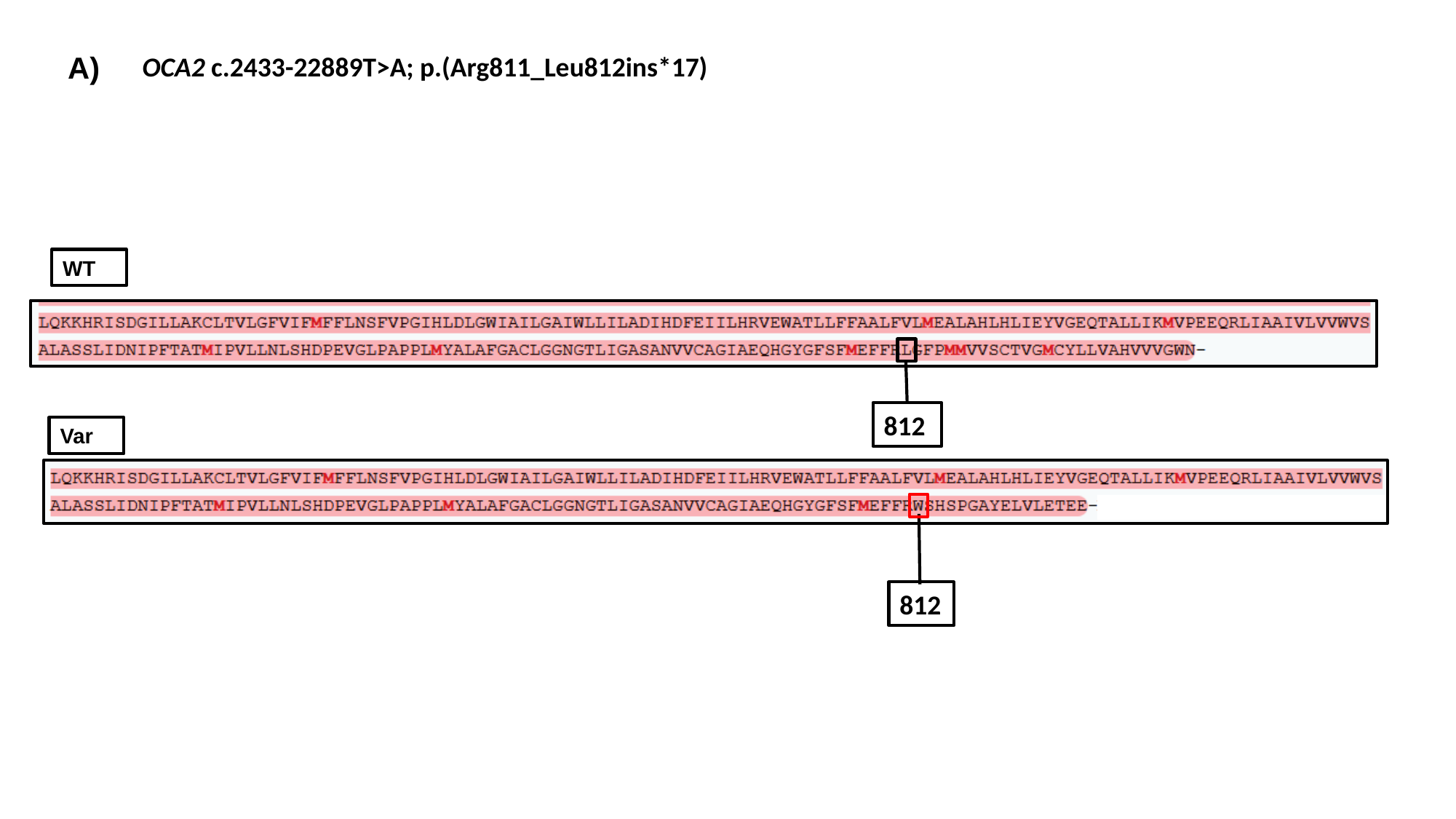

A)
OCA2 c.2433-22889T>A; p.(Arg811_Leu812ins*17)
WT
812
812
Var

## Slide 2
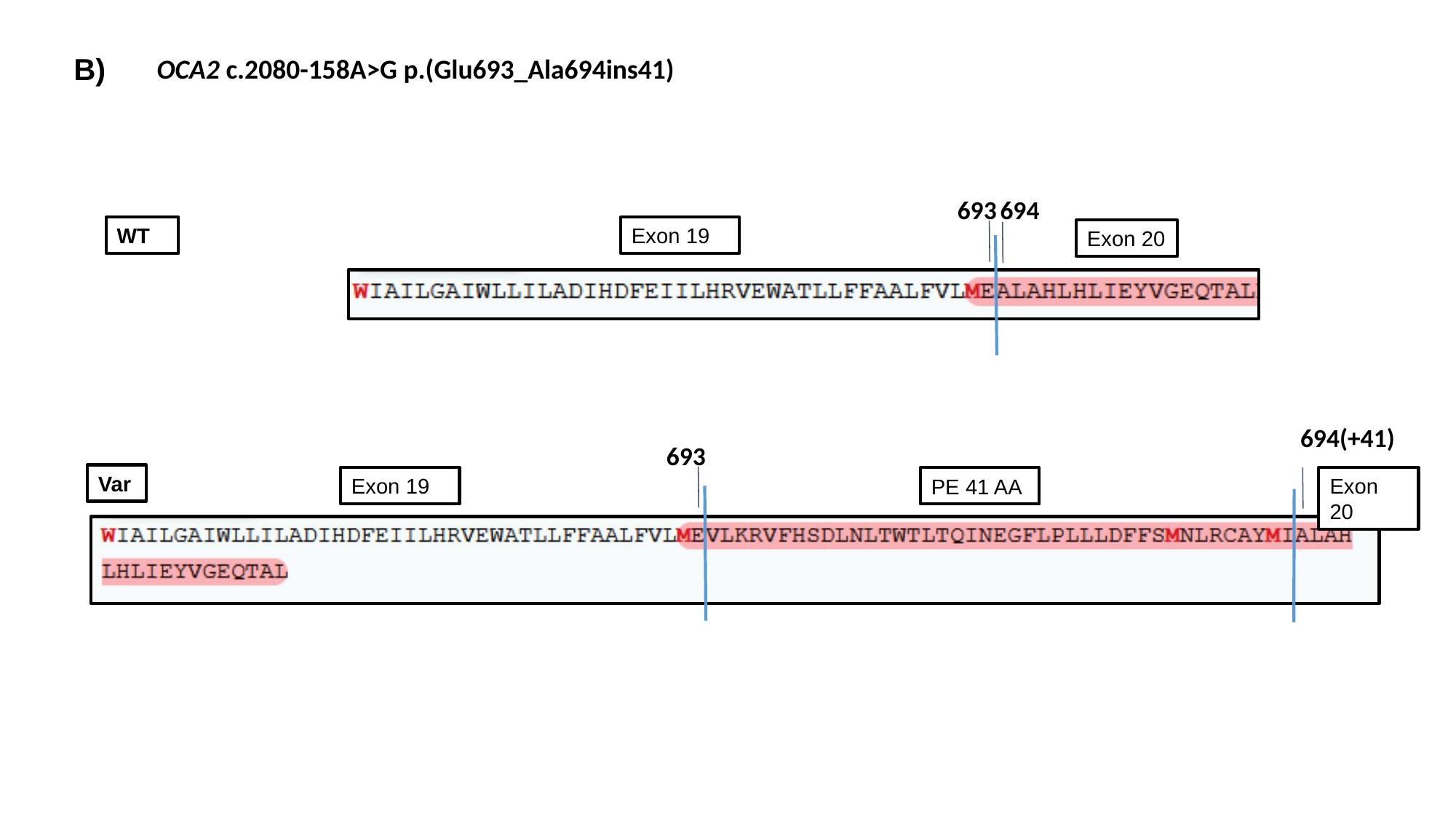

B)
OCA2 c.2080-158A>G p.(Glu693_Ala694ins41)
693
694
WT
Exon 19
Exon 20
694(+41)
693
Var
Exon 19
Exon 20
PE 41 AA

## Slide 3
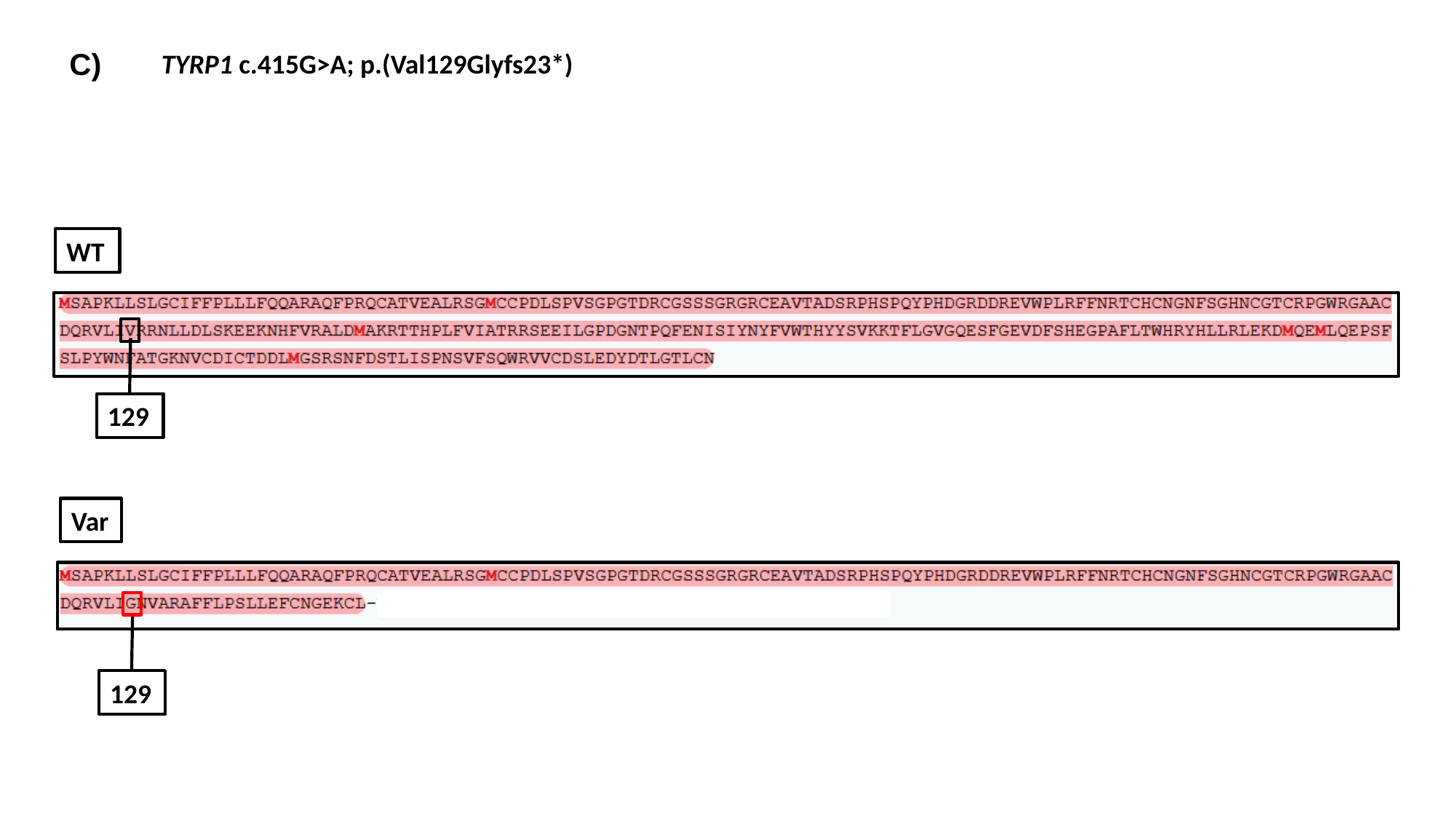

C)
TYRP1 c.415G>A; p.(Val129Glyfs23*)
WT
129
Var
129

## Slide 4
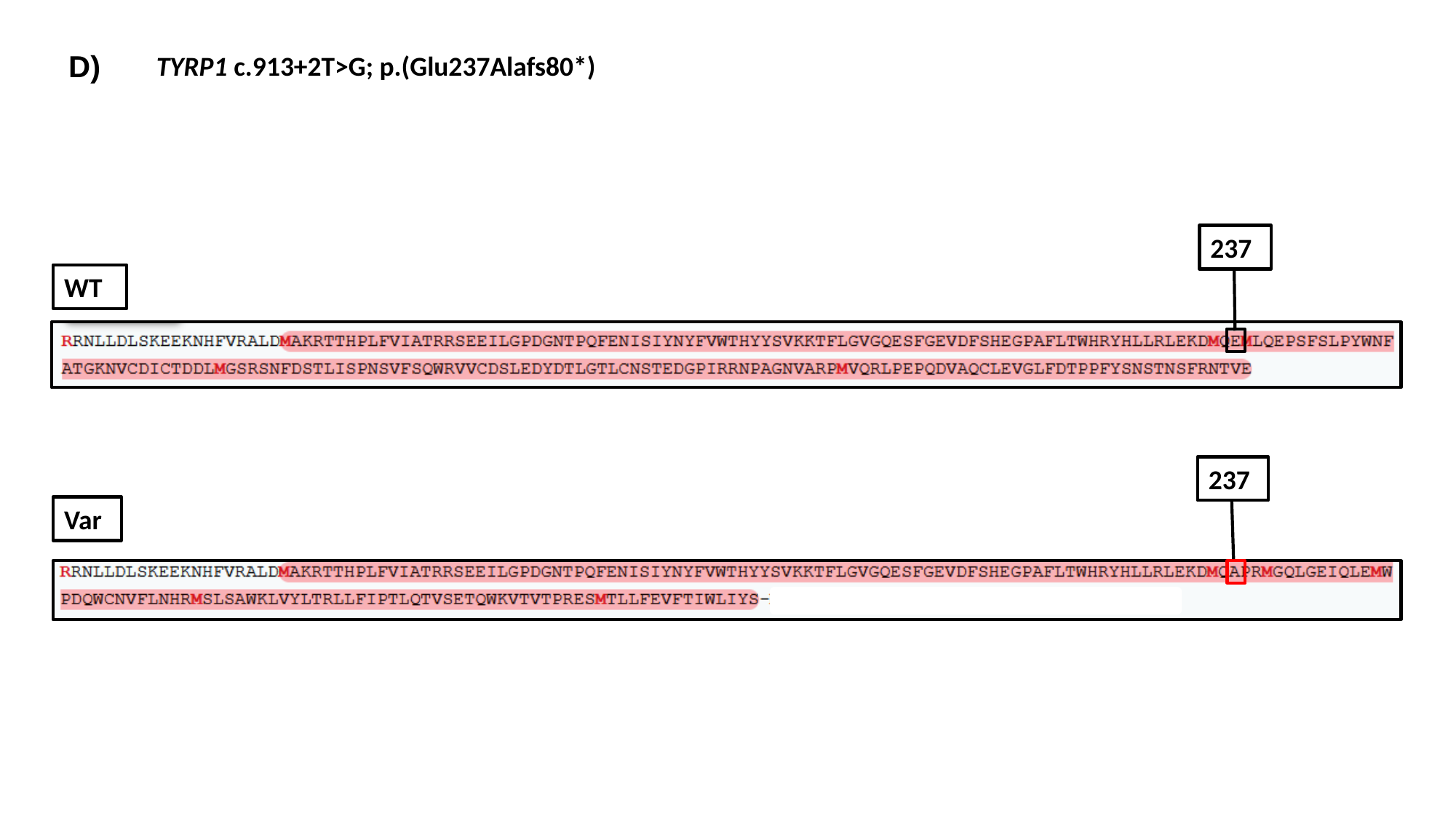

D)
TYRP1 c.913+2T>G; p.(Glu237Alafs80*)
237
WT
237
Var

## Slide 5
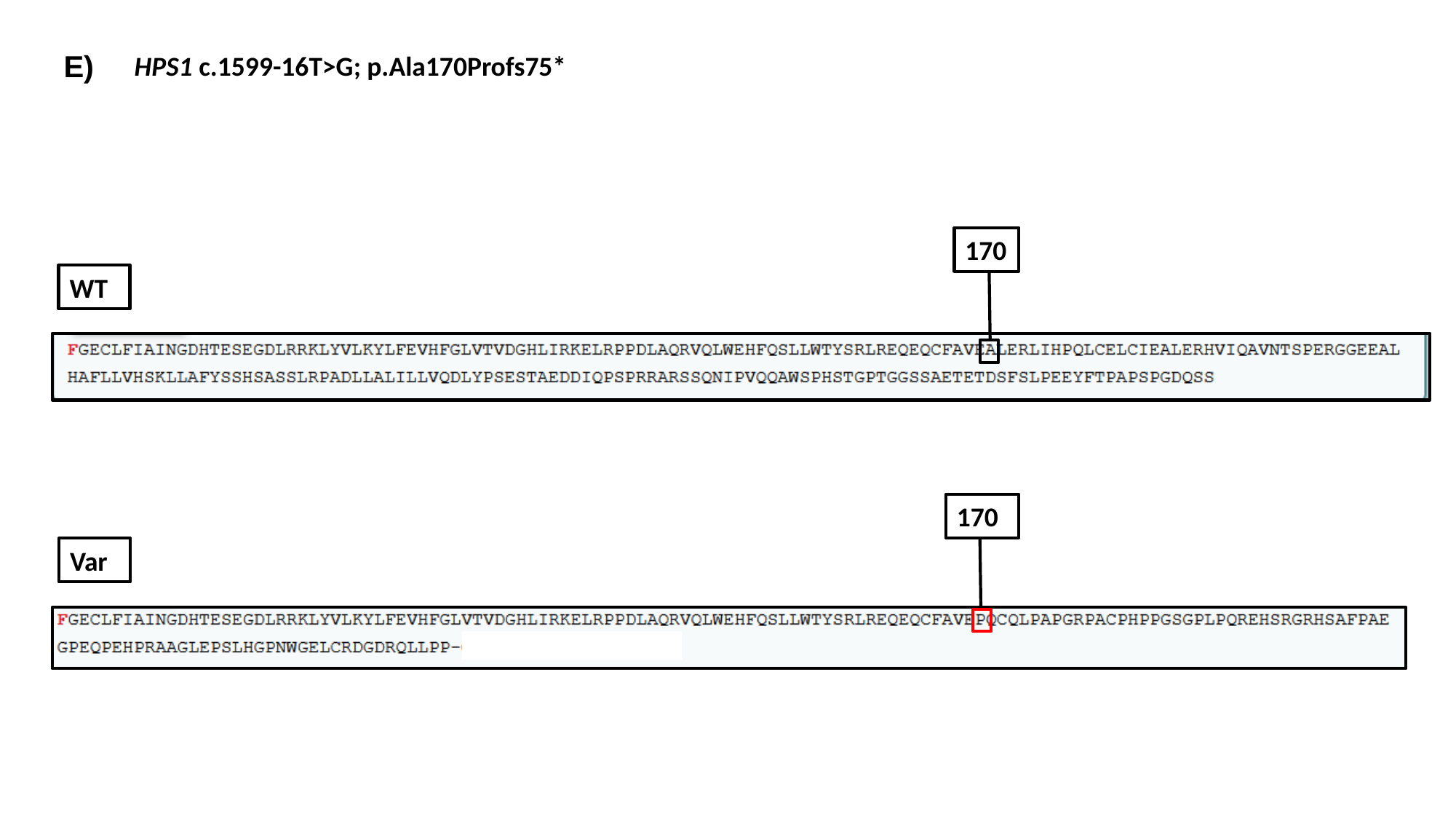

E)
HPS1 c.1599-16T>G; p.Ala170Profs75*
170
WT
170
Var
