# Supplementary material for: Functional Characterization of Splice Variants in the Diagnosis of Albinism
Source: Int J Mol Sci. 2024 Aug 8;25(16):8657. doi: 10.3390/ijms25168657 (PMC11355033; doi:10.3390/ijms25168657)
Supplement: Supplementary file 1 [file ijms-25-08657-s001.zip › Supplementary Figure S2.pptx]

## Slide 1
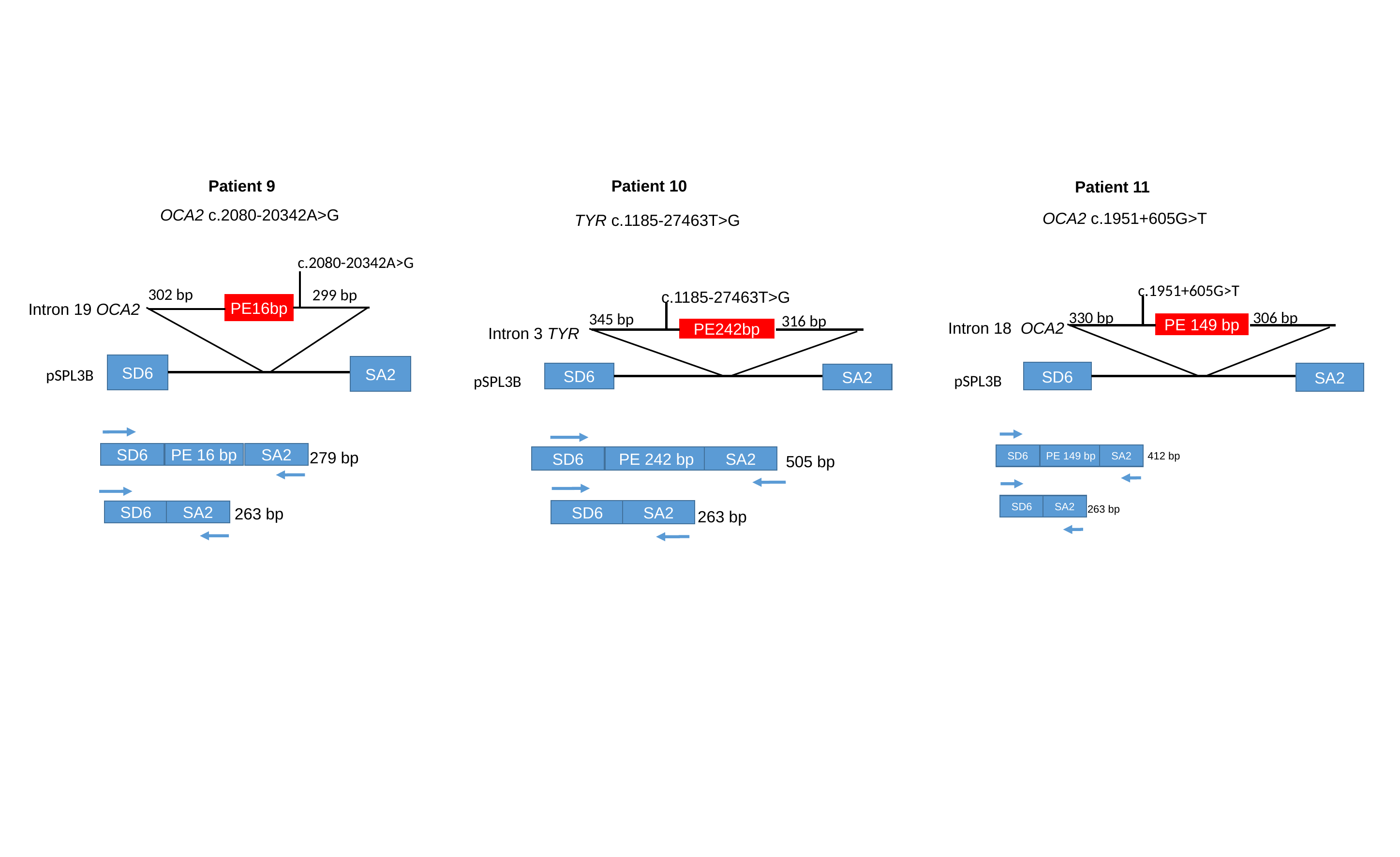

Patient 9
Patient 10
Patient 11
OCA2 c.2080-20342A>G
OCA2 c.1951+605G>T
TYR c.1185-27463T>G
c.2080-20342A>G
302 bp
299 bp
PE16bp
Intron 19 OCA2
SD6
SA2
pSPL3B
c.1951+605G>T
330 bp
306 bp
PE 149 bp
Intron 18 OCA2
SD6
SA2
pSPL3B
c.1185-27463T>G
345 bp
316 bp
PE242bp
Intron 3 TYR
SD6
SA2
pSPL3B
SD6
PE 16 bp
SA2
279 bp
SD6
SA2
263 bp
SD6
PE 149 bp
SA2
 412 bp
SD6
SA2
263 bp
SD6
PE 242 bp
SA2
505 bp
SD6
SA2
263 bp
