# Supplementary material for: Functional Characterization of Splice Variants in the Diagnosis of Albinism
Source: Int J Mol Sci. 2024 Aug 8;25(16):8657. doi: 10.3390/ijms25168657 (PMC11355033; doi:10.3390/ijms25168657)
Supplement: Supplementary file 1 [file ijms-25-08657-s001.zip › Supplementary Figure S3.pptx]

## Slide 1
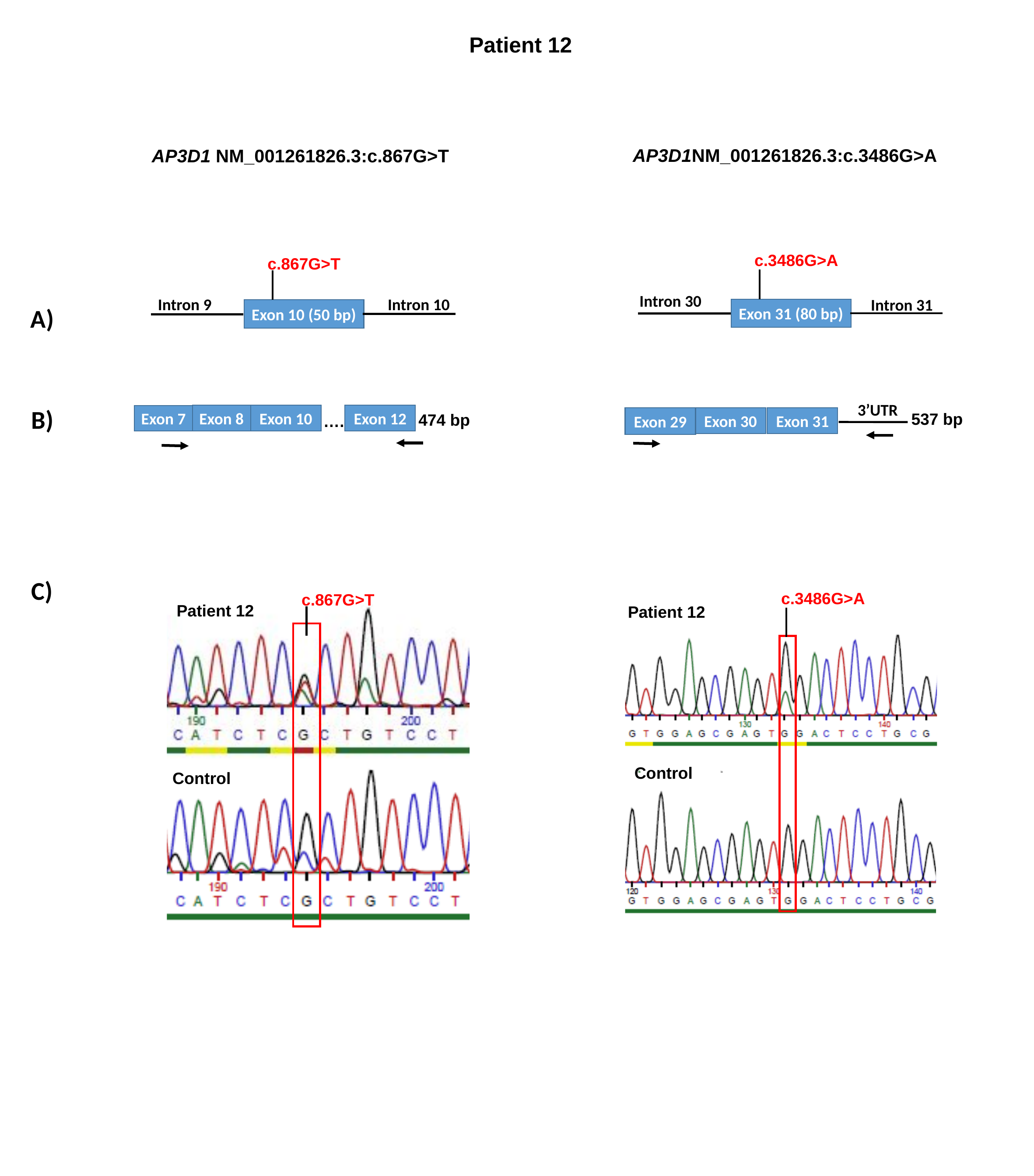

Patient 12
AP3D1NM_001261826.3:c.3486G>A
AP3D1 NM_001261826.3:c.867G>T
c.3486G>A
Exon 31 (80 bp)
Intron 30
Intron 31
c.867G>T
Intron 9
Intron 10
Exon 10 (50 bp)
A)
3’UTR
537 bp
Exon 29
Exon 31
Exon 30
B)
Exon 10
Exon 8
Exon 12
Exon 7
474 bp
….
C)
c.3486G>A
c.867G>T
Patient 12
Control
Patient 12
/
Control
